# Nursing Students' Perceptions of Nursing Metaparadigms: A Phenomenological Study

**DOI:** 10.1097/jnr.0000000000000311

**Published:** 2019-09-20

**Authors:** Ayse DELIKTAS, Oznur KORUKCU, Ruveyde AYDIN, Kamile KABUKCUOGLU

**Affiliations:** 1 MSc, RN, Research Assistant, Faculty of Nursing, Department of Obstetrics & Gynaecological Nursing, Akdeniz University, Turkey.; 2 PhD, RN, Associate Professor, Faculty of Nursing, Department of Obstetrics & Gynaecological Nursing, Akdeniz University, Turkey.; 3 PhD, RN, Professor, Faculty of Nursing, Department of Obstetrics & Gynaecological Nursing, Akdeniz University, Turkey.

**Keywords:** health, nursing, metaparadigm, paradigm, students

## Abstract

**Background:**

The paradigm is a vital concept steering the development of a scientific discipline. Paradigms that shape the education, research, and practice steps of a discipline are defined as metaparadigms.

**Purpose:**

The purpose of this study was to explore the perception of nursing students regarding metaparadigms in nursing at Akdeniz University in Antalya, Turkey.

**Methods:**

This was designed as a descriptive phenomenological study, and data were collected from 13 fourth-year students who were chosen via a purposeful sampling method and interviewed face-to-face using a semistructured format. Data were analyzed using the data analysis steps of Giorgi, who is an expert in descriptive phenomenological studies.

**Results:**

Most of the participants in this study associated nursing with humanism. Some of the participants stated that a fundamental building block of the nursing profession is conscience, whereas others stated that nursing is a way to touch people's lives and is now regarded as a professional practice. It has been reported that students have difficulties identifying metaparadigms in nursing and that they believe that human beings have the potential to widen their horizons with wisdom and social skills. According to the participants, the health metaparadigm refers to the harmony between human beings and their environment and to the autonomy of the individual. Moreover, the participants emphasized the well-being of individuals.

**Conclusions/Implications for Practice:**

The participants in this study characterized humans with well-being, transcendence, adaptation and interaction skills with their environment, and harmony beyond physiological mechanisms, which was considered to be promising for the perception of future nurses. It is recommended for nursing educators to enable their students to raise their professional awareness and to internalize professional values.

## Introduction

Paradigm is a vital concept in the philosophy of science that has far-reaching influence on contemporary empirical studies (Fawcett, 2000). Kuhn (2017) defined paradigm to identify study models that shape scientific activities and the progress of scientific knowledge (Smith & Parker, 2015). Kuhn argued that scientific paradigms determine the study questions, interview methodology, data collection methods, and interpretation of study results. Paradigms shape individual behaviors, influences of social phenomena, and attitudes of professional groups. Therefore, paradigms serve as a guide for professional education, philosophy, morals, and ethical principles (Özkan & Akduran, 2014).

Metaparadigms, or dominant paradigms, map out general parameters of a scientific discipline and focus on scientific efforts. Metaparadigms may include several concrete and specific paradigms for researchers (Meleis, 2011). Clarifying metaparadigms and their fundamental thesis statements facilitates a deeper understanding of the attitudes of professionals and provides a better appreciation of the scope of scientific studies (Kim, 2000).

Nursing metaparadigms were first classified by Fawcett (1978) into the following categories: person, environment, health, and nursing. The human factor metaparadigm refers to individuals in a definite culture, family, and society. The environment metaparadigm characterizes all regional, national, and global cultural, social, political, and economic conditions related to human health. The health metaparadigm defines processes of life and death. The person metaparadigm describes the nursing profession, nursing practices, and nursing objectives and results (Bahramnezhad, Shiri, Asgari, & Afshar, 2015; Fawcett, 2000). In addition to these four metaparadigms, different versions of the nursing metaparadigm have been offered. For instance, Watson considered nursing care to be the core indicator of nursing practice and suggested nursing care as the fifth metaparadigm (Watson, 2008).

In recent years, the conceptual framework of nursing has been used to design nursing curricula. Conceptual frameworks are also regarded as models to construct nursing values and concepts (Alligood, 2014). It is utterly important for the professional development of nursing students that they integrate their clinical experiences into theoretical knowledge. An insight into nursing metaparadigms will certainly enable nursing students to cope with the challenges that face them in developing their professional identity (Lee & Fawcett, 2013).

In a recent study, Fawcett (2000) taught “Transition to Professional Nursing” to nursing students and analyzed their feedback with regard to nursing metaparadigms. The participants noted that raising awareness toward nursing paradigms provided a framework for the nursing profession and an intellectual recognition of the fundamentals of nursing while reducing burnout (Fawcett, 2000). A participant further stated that nurses were still a member of a professional group, even when they ignored the significance of nursing paradigms (Lee & Fawcett, 2013). In the same study, another student, on the other hand, admitted that “I have never heard of nursing paradigms before. But now it makes me feel even more dedicated to my professional duties when I think over my nursing philosophy and nursing experiences.”

Nurses' perceptions of nursing metaparadigms and their awareness of professional concepts will eventually influence their professional development (Fawcett, 2000). Awareness of professional identities and values is established during professional education. Therefore, it is of utmost importance to investigate nursing students' perceptions of fundamental nursing values and metaparadigms. Accordingly, this study aims to analyze nursing students' perceptions of the four basic nursing metaparadigms (person, human factor, health/illness, and environment).

## Methods

### Research Design

This study was designed as a descriptive, phenomenological study. The main point of phenomenological studies is to unveil participants' personal experiences and perceptions of concepts (Creswell, 2014). Researchers use descriptive approaches to attempt to describe very carefully the experiences being lived through, and once raw data have been obtained, a thorough phenomenological psychological analysis of the data takes place within the perspective of the phenomenological psychological reduction (Giorgi, 2012). Students are provided with metaphors to signify person and health/illness metaparadigms, which help exemplify the unknown with known examples so that sophisticated ideas may be conveyed and identified more easily, eventually enabling the participants to symbolize their experiences with a single image (Schmitt, 2005).

### Participants

The study sample included fourth-year students at Akdeniz University Nursing Faculty in the 2016–2017 academic year. Purposeful sampling was used to recruit participants. The inclusion criteria of this study were as follows: attending the nursing school as a fourth-year student and being interested in nursing philosophy, being an active member of the Turkish Student Nurses Association, and having completed taking History and Deontology of Nursing and Fundamentals of Nursing courses.

In the Turkish nursing education system, metaparadigms are taught only in History and Deontology of Nursing and Fundamentals of Nursing courses. Thus, students in these courses tend to be more aware of these metaparadigms. Otherwise, eligible students who did not successfully pass these courses were excluded from participation.

The sample size in qualitative studies is determined by data saturation, which is marked by repetitive input from newly added participants (Morse, 2015). Data saturation was reached in this study after 13 participants had been interviewed. The participants included only two male students.

### Data Collection

The participants were interviewed in the meeting hall of the department using an in-depth interview method, which is the most frequently used data collection tool in phenomenological studies (Creswell, 2014).

A personal information form and a semistructured questionnaire, both designed by the researchers, were used in the interviews. The researchers consulted with the experts while designing the semistructured questionnaire. All of the interviews were audio recorded. The interview room was set up for the interviews, and a notice was put at the door. The purpose of the research was explained to the participants by the researchers before their interview session. As suggested by Moustakas (1994), the researchers began the interview with an ice-breaking question like “Would you like to talk about concepts that are significant for nursing practice?” to test their awareness of metaparadigms and to encourage participants' participation in the interview. Afterward, the interview continued with main items and subitems, if necessary, to help the participants express their perceptions and opinions (Table 1). The interview lasted approximately 35–45 minutes.

**TABLE 1.**
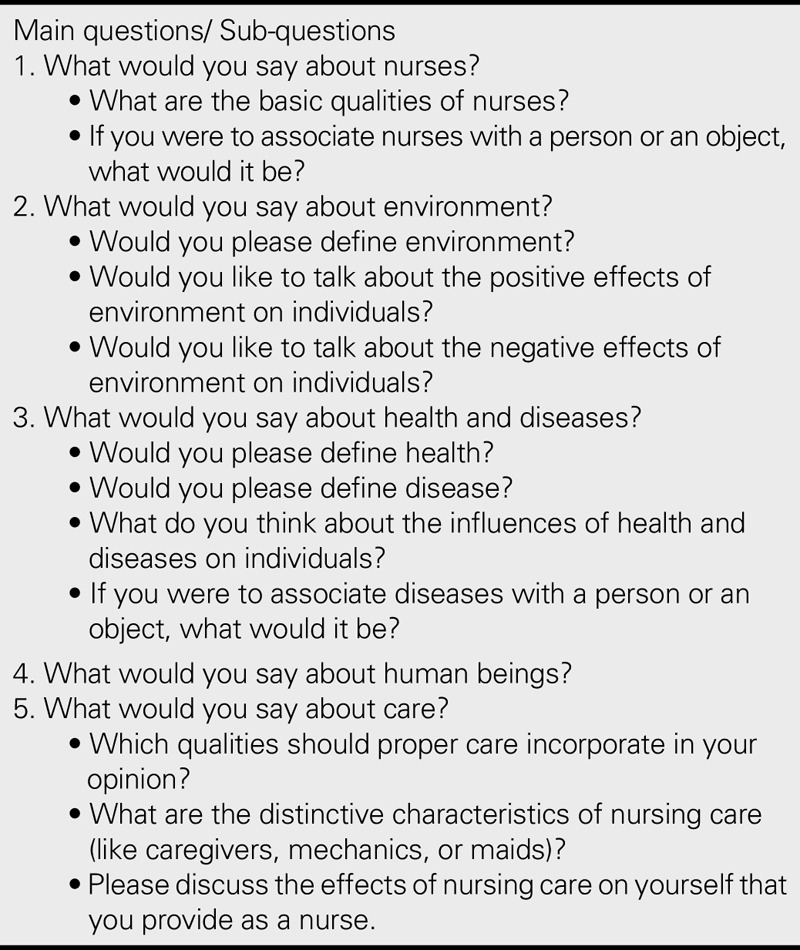
Research Questions

### Rigor and Trustworthiness

Lincoln and Guba (1985) proposed that certain criteria, including credibility, transferability, dependability, and confirmability, must be sought in qualitative studies to test reliability and validity. Rigor and trustworthiness were achieved through individual interviews using a semistructured interview form to ensure that the participants were asked a similar range of questions. The researchers, who interviewed the participants and who were not teachers of the participants, were doctoral candidates who held master of science degrees in nursing, had training in qualitative research, and had published more than 10 nursing articles each. Data were coded by two independent researchers (a professor and an assistant professor who were experts in nursing education and qualitative research). For validity, all interview data were transcribed without adding any comments for dependability criterion, and data coding was carried out by two researchers individually. During the coding process, researchers exchanged and discussed their opinions regarding the compliance of codes obtained from data that were repeated. For confirmability (verifiability), a comprehensive interview form and the final version of thematization were evaluated by an expert person.

### Data Collection

Study data were collected using a 6-item personal information form that gathered information on the descriptive characteristics of the participants and a 15-item semi-structured interview form that gathered information on the participant’s perception of nursing metaparadigms (Table 1). The suitability of the questions in the semi-structured interview form was assessed by a professor and a doctor working in the field of nursing education. Individual, in-depth interviews were conducted with three students to test the validity and reliability of the semi-structured interview form. The researcher rearranged interview questions according to the results of the pilot study. The question “Do you think that your life changed after you encountered nursing paradigms?” was asked to start the interview.

### Data Analysis

The interviews were recorded on camera and with a voice recorder. The author analysed the data obtained from the study through interview outputs using a written solution and then determined the themes. The study data were analysed in four basic steps, as suggested by Giorgi (2012) for descriptive phenomenological studies. According to this approach a phenomenological attitude, i.e., looking at objects from the perspective of how they are experienced, allows a search for the essence of the phenomenon using free, imaginative variation to reveal why the object makes a specific example of the phenomenon. The procedure consists of the following steps: (a) total impression—from chaos to themes; (b) identifying and sorting meaning units—from themes to codes; (c) condensation—from code to meaning; (d) synthesizing—from condensation to descriptions and concepts (Giorgi 2012; Malterud, 2012).

### Ethical Considerations

Ethical approval was obtained from the ethical committee of the Akdeniz University Clinical Studies Board of Ethics, confirming that this study follows international standards and the principles adopted in the World Medical Association Declaration of Helsinki (approval No. 15, August, 2017). The Declaration of Helsinki was signed by all researchers. Before commencing the study, the researchers obtained permission from the dean of the Faculty of Nursing. The contact information for the students who met the inclusion criteria was obtained. Oral and written consent was obtained after the students who were targeted for participation were informed about the study. Before each interview session, the participants were informed that interviews would be recorded by camera and voice recorder. Furthermore, they were notified that their voice recordings would not be used for any purpose other than the study. Then, the permission of the participants was obtained. The participants were informed that they were free to leave the room and quit the interview anytime they wished and that their decision to participate or not carried no risk to them.

## Results

The age of participants ranged from 20–23 years old; a majority were females (*n* = 11); and most stated having a moderate interest in philosophy (*n* = 9).

The perceptions and opinions of student nurses regarding the four basic metaparadigms of nursing were thematically grouped and analysed (Table 2).

**TABLE 2.**
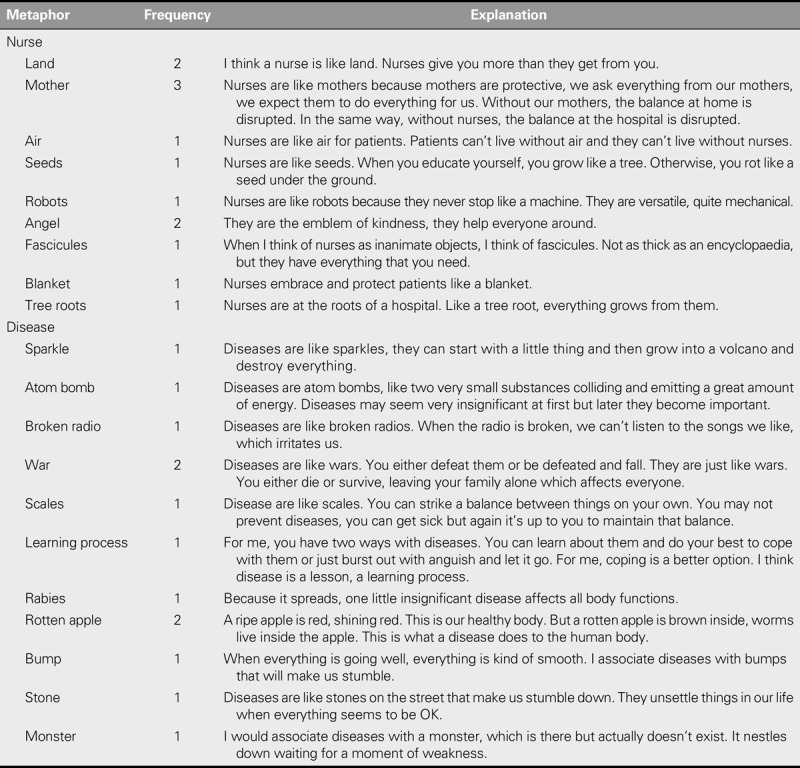
Metaphors That Nursing Students Associated With Nursing and Disease Metaparadigms and Their Explanations

### Theme 1. “Person” Metaparadigm from the Perspective of Participants

The thoughts of participants about the person metaparadigm were investigated under five sub-themes, including: person as a wise being, person as a potentially self-transcendent being, person in interaction with his/her environment, person as a socially adaptable being, and person with management skills.

#### 1. Person as a wise being

The student nurses defined person in a variety of ways. One participant expressed that it was difficult to define what a person is, as humans cannot be easily generalized. However, she said, the most distinguishing characteristic of personhood is the ability to think.

*Human beings are difficult to define and impossible to stereotype. Even the most basic physiological needs may vary from individual to individual. Some sleep for long hours, some less; some eat a lot, some only a little; some show their love easily, some can't … I believe words are not enough to define human beings. Because each human being is a distinct world and needs individual attention. But, I believe their most distinguishing characteristics is their ability to think.* (S6)

One of the participants remarked that a person is special for his/her cognitive skills:

*I think what makes human beings special is their ability to think. Human beings, with the help of their thinking skills, can make their own decisions, make their own choices, and face the consequences of their decisions.* (S3)

Another participant expressed that people can only exist as social beings due to their ability to think:

*I think the best part of being a person is having the ability to think. With that ability, a person can create, perform scientific research, and produce art. People exist as social beings to the extent of their thinking ability. Isn’t this what distinguishes them from other living beings?* (S8)

Another student thought that the ability to think and reason is the greatest gift for humanity:

*I think the potential to think and reason is the greatest gift for humanity. A thinking human being has the ability to make decisions, make their own choices, and face the consequences of their decisions. Thinking is the only humane way for an individual to lead a free life endowed with fraternity and civil rights.* (S9)

#### 2. Person as a potentially self-transcendent being

A participant expressed the belief that people are not machines but potentially self-transcendent beings:

*A person is not only a machine composed merely of eyes, heart, veins, and muscles. A person is their emotions, spirit, and a sum of pieces. What is more, people are extraordinary creatures who transcend themselves with aging, proving their potential to go beyond and their ability to widen their horizon.* (S10)

Another participant posited that people have, in fact, an ability to go beyond their potential but that they may also be full of remorse when they are unable to fulfil their potential:

*People are amazing and they can do whatever they want when they want it deep in their heart. They have hobbies and skills. They listen to music, play musical instruments, paint, do all kind of sports, and dance. They find hobbies to enjoy their time among their daily routines. But there are also a lot of people who don’t care about themselves, live their life in stress, and don’t appreciate what life brings to them.* (S11)

#### 3. Person in interactions with his / her environment

Most participants defined people as social beings and stated that people should interact with their social environment:

*People are social beings, and they should interact with their environment for a happy life. I think that you can’t be happy if you are all alone and isolated from others and the environment. People must be in a relationship with their friends, family members, other people, and even other living beings.* (S12)

#### 4. Person as a socially adaptable being

One of the participants identified people in the context of their ability to adapt to changing conditions. Regardless of their material conditions, people can adapt to new conditions:

*I believe that a characteristic of people is being able to adapt to different conditions. People are so amazing that they can eventually adapt to new conditions despite having difficulties at first. For example, someone who lost his/her whole family or who became disabled for the rest of his/her life after an accident, life can be difficult at first. Sometimes it takes years to get used to their new life, or they might even receive psychological support. But, in the end, they learn how to deal with their new life. Because they have no other option but to learn how to struggle with changes in their lives.* (S7)

#### 5. Person with management skills

Some participants agreed that one peculiar quality of personhood is having management skills, although these may cause some problems at times:

*Although it may occasionally create problems, I believe that one of the most significant characteristics of a person is the ability to manage other people. Indeed, they manage not only other people but also themselves.* (S1)

Another stated that:

*I believe that people are capable of educating each other, with the potential to develop and grow and manage their environment and other people.* (S13)

### Theme 2. “Health/Illness” Metaparadigm from the Perspective of Participants

The perceptions of the participants regarding the health/illness metaparadigm were categorized into the sub-themes of stability/instability, well-being/deterioration in well-being, and independence/dependence. The participants were also asked what metaphors they would use for the illness meta-paradigm and their answers were sparkle, atom bomb, broken radio, war, scales, rotten apple, learning process, rabies, monster, and bumper (Table 2).

#### 1. Stability/instability

Some participants associated health and illness with stability, defined health as the harmony between human beings and their environment, and defined illness as the disturbance of well-being:

*I think that the human body works in harmony. Illness is the disturbance of this harmony. For instance, mothers maintain harmony in the house. When they get sick, all harmony is disrupted and things go wrong. In the same way, when there is a health problem in the human body, things go wrong and all systems are negatively affected.* (S11)

Another student compared human health to wheel alignment on cars. S/he stated that the slightest instability disrupts balance in the human body and eventually causes illnesses.

*The human body is like a machine, and health is a state of balance. People are healthy to the extent that they maintain that balance. Even if the smallest part of the human body doesn’t work in harmony with other parts, it causes instability in human health.* (S13)

#### 2. Well-being/ deterioration in well-being

A majority of participants identified health and illness with well-being. They defined health as well-being of the body and soul, and interpreted illness as a deterioration in well-being:

*Health means feeling well. I think you are healthy as long as you feel well. I mean, you are healthy when your body works normally. For instance, if a person with high blood pressure feels well, they are healthy. I believe that being diagnosed with high blood pressure doesn’t make one a sick person.* (S7)

Another student who characterized health with well-being stated that:

*In Turkish culture, when people ask how you feel, people answer “I am good, I am healthy” instead of just saying “I am feeling well”. I mean, health is equal to feeling well in our culture. People are healthy as much as they feel well, I think.* (S1)

#### 3. Independence/dependence

One of the participants defined health as the capacity to cover one’s own needs independently and illness as being dependent on others:

*Health is the capacity to cover one’s own needs, whereas illness is not. In other words, we can’t say one is healthy if they need help from other people to cover their needs.* (S5)

### Theme 3. “Environment” Metaparadigm from the Perspective of Students

Most participants defined environment as almost everything that is around a person:

*I can’t find a word to define environment. Everything, abstract or concrete, is your environment. Having my hand on that desk now, the heat of the weather I feel, having eye contact with you at this very moment - all are components of our environment ... When I talk to my friends, what they make me feel is abstract. Feeling comfortable or anxious here and now is an example of such feelings.* (S4)

Another participant associated environment with happiness:

*Any place where people feel happy or unhappy and in which they interact is their environment.* (S9)

While some students referred to environment as physical environment, others defined the social environment and psychological environment. The main theme of environment was examined under three sub-themes: physical environment, psychological environment, and social environment.

#### 1. Physical environment

Some participants in the study stressed the healing effect of environment and they underlined certain qualities of environment such as warmth, light, comfort, cleanliness, and silence to have a healing effect:

*A room should be well-lighted with enough windows. The walls may be painted in colours. Each clinic has a certain order of its own. For instance, rooms in paediatric clinics must have balloons and ornaments. The physical environment should make people feel good.* (S3)

#### 2. Psychological environment

Some participants mentioned several qualities of environment that have a healing effect, including being kind, sincere, good-humoured, and trustworthy.

One participant stated that:

*When one talks about environment, it is not only furniture and rooms, of course. Psychological environment is also important. Family life, friends, nurses, … these are all your environment. A nurse should provide quality care, be good-humoured, and ask patients how they feel, which is very precious for a patient. This is what comprises a patient’s psychological environment.* (S2)

#### 3. Social environment

Some participants approached the environment from a social perspective:

*One’s environments isn’t only made of mechanical components. I mean, air, water, sun, or houses … They don’t define one’s environment. People around you are your social environment. Nurses, in a hospital environment, are a part of patient’s social environment. Nurses affect patients and patients affect nurses in turn.* (S13)

*The social environment for a patient should be warm and sincere. People around the patient should be good-humoured. For example, patients must feel like they are at home rather than in a hospital room. We, the nurses, must work to ensure this.* (S8)

### Theme 4. Nursing Paradigm from the Perspective of Participants

The participants associated the nursing metaparadigm with humanism, touching the lives of individuals and professional identity. Moreover, when they were asked “If you were to associate nurses with a person or an object, what would it be?”, their answers included land, air, seed, robots, angels, fascicules, blanket, and tree roots (Table 2).

#### 1. Humanism

A majority of participants identified nursing with humanism. One participant who believes that one should learn to love themselves before they serve others stated that:

*I think humanism is at the heart of nursing. A good nurse should love himself/herself in order to love other people. How can you expect a person to help other people heal if s/he doesn’t love him/herself in the first place?* (S1)

Another expressed that the humanism factor is frequently emphasized in their courses and said that becoming a good nurse is only possible by loving people:

*In all our courses and practices in the department, we are reminded of the significance of a humanistic approach before teaching the theory of nursing. Humanism is basically loving and understanding people. I learned in the Ethics of Nursing course that the first and foremost human right is the right to live and that my duty is to ensure that my patients’ lives take a humane course to the last moment. I believe that the secret of success in nursing lies in humanism.* (S2)

Furthermore, some participants pointed out that conscience should be one of the key components of nursing.

*I think students should be taught the merits of conscience. I believe that people without mercy in their heart can’t help other people. A good nurse is also a good person and, in my humble opinion, only people with a conscience can be good people. As the number of nurses with a conscience and with mercy in their hearts increases, the nursing profession will no longer be limited to giving injections, aspirating patients, and checking blood pressure.* (S3)

#### 2. Touching the lives of people

One of the participants stated that nurses should touch patients not only with their hands or eyes but also with their hearts, and defined nursing as a way of touching people’s lives:

*Nursing is touching people’s lives. The nursing profession is based on patient care, which is indeed very precious in itself. I think nursing is the ability to touch people’s lives not with your hands or eyes but with your heart. Any individual can provide care for sick people but it is a special skill to take care of a person in need with your whole heart. That is what makes nursing a special and meaningful profession - touching a patient’s life with your heart.* (S4)

Another participant stated:

*I love nursing. We are asked to take care of two patients during our clinical practice. I meet two new families every week and become a part of these families. I touch their lives and they touch mine. I think that is the best part of nursing - getting involved in new lives.* (S5)

#### 3. Professional identity

Some participants associated nursing with professional identity. A student who defied the angel stereotype highlighted that:

*I am mad at people who define nurses as angels without wings, as sacred, as a sacrifice, as white angels ... Hearing such clichés disinclines me in my professional ambitions. It makes me sad when people only identify nurses as white angels, when nurses actually strive to provide a quality of care that is exactly what their patients need. People must understand what a professional means and demand quality care.* (S6)

Another participant stated the belief that nursing should be based on professional principles:

*Like any other service business, nursing should be based on professional practice. I can’t think of any nursing care without professional principles. I believe that there is need for a professional practice to maintain mutual respect between nurses and patients and their relatives and to provide quality care.* (S7)

## Discussion

Paradigms are indispensable for the development of the nursing profession on a scientific basis (Mc Evan & Will, 2006). Therefore, nursing students who will carry the nursing profession into the future are expected to raise awareness of the metaparadigms of nursing and build up their own nursing philosophy upon these paradigms. It is obvious that providing students with the fundamental principles of nursing and special skills to define nursing care requires more than only teaching them the theory of nursing (Mc Evan & Will, 2006).

Undergraduate nursing students take theoretical and practical courses for four years (total 240 ECTS credits) in the university where this study was conducted. In their first year in the department, they are taught basic principles of nursing in the Fundamentals of Nursing I course. Besides, they are also given the fundamentals of effective communication and of the nursing profession in the Interpersonal Relations course. In their second semester in the first year, students establish close contact with patients, first in labs and then in clinical practice in the Fundamentals of Nursing II course. Furthermore, the students are offered elective theoretical courses such as the History and Deontology of Nursing, Professional Communication, Ethics of Medical Care, and Communication Skills. In their eighth semester, students have a chance to put theoretical education into practice under the supervision of their mentor nurses and clinical lecturers. It is of utmost importance to enable nursing students to define the nursing profession on their own and to develop their own perspectives on nursing values before graduation.

A humanistic approach has a far-reaching influence on an individual’s attitudes and behaviours (R⊘nnow-Rasmussen, 2008). Moreover, a humanistic approach brings out a humble and merciful attitude (Pembroke, 2006). Nurses who cherish their patients will certainly enjoy their jobs and have a successful professional career (de Vries, 2004; Stickley & Freshwater, 2002). In this study, participants widely agreed that nurses should love people before helping them. In addition, Watson (2007), suggested that nursing care is based on universal humanistic values. In this study, the participants expressed that humanism is a vital aspect of nursing and that humanism is, at its core, loving people. Furthermore, it is noteworthy that the participants believed that nurses should first and foremost touch their patients not with their hands or eyes but with their hearts. It encouraging that the student nurses in this study regarded professional identity, humanism, and mercy as the fundamental values of nursing.

The primary focus of nursing is the human factor (Rodgers, 2005). Therefore, it is necessary to understand people and their needs in order to provide the best care possible (Mc Evan & Will, 2006). Leininger (1997) emphasized the necessity of care to maintain health and individuality and added that care was different from treatment. The fundamental values of nursing should be integrated into the nursing curricula in both theoretical courses and clinical practices (Rodgers, 2005). The health metaparadigm plays a crucial role in nursing education and practice and has been widely institutionalized. In a study on the perceptions of nursing students toward health metaparadigm conducted in Sweden, it was found that students associated health with physical and mental well-being and that they considered that individuals were primarily responsible for promoting their health. In addition, these students believed that promoting health is also deeply related with maintaining a balance between diet, activities, work, and leisure. Furthermore, having healthy relations with family and friends is a prerequisite for having good health. A student in the study also stated that individuals may feel healthy despite having relatively bad health (Skär & Söderberg, 2016).

Hernandez (2009) carried out a similar study and asked nursing students to write down their opinions toward the person, health, human, and environment metaparadigms after having participated in a course on nursing theory. They were also asked to work in study groups and to reflect on their own nursing philosophy through case studies. The assessment of case studies illustrated that these studies provided students with a critical perspective and helped them better grasp the core of nursing theories. Consequently, it was suggested that teaching nursing metaparadigms enhanced the acquisition of professional values.

Nursing practice is not only putting theory of nursing into practice but also a professional field in which new ideas are produced and tested to develop nursing knowledge (McEvan & Will, 2006). In addition, metaparadigms pave the way for students to develop a critical perspective and to become a professional member of an academic discipline. Thus, nursing education should be designed holistically to include nursing paradigms to assist nursing students understand the basic needs and experiences of individuals (Eriksson, 2007).

Thus, student nurses should raise awareness toward nursing values from the onset of undergraduate education. Nursing students, consciously or unconsciously, develop their own nursing definitions throughout theoretical and practical education. Therefore, nursing educators should design programs that pave the way for professional nursing education by encouraging the more active participation of nursing students.

### Conclusions and Implications for Practice

This study found that nursing students focused not only on the physical but also on the emotional, spiritual, social, and environmental aspects of nursing metaparadigms. Most of the students in the study associated nursing with humanism. Previous studies have also reported that students have difficulties in identifying nursing metaparadigms and that they believe that humans have the potential to widen their horizons with wisdom and social skills. In this study, most of the participants were aware of the meaning of nursing and their perceptions toward nursing paradigms were in line with mainstream nursing theories. However, it is quite challenging for nursing students to connect nursing paradigms with nursing practice, which eventually causes discrepancies between theory and practice. Therefore, nursing students should be encouraged to internalize metaparadigms through courses or study groups in order to successfully comprehend the significance of metaparadigms and establish strong ties between nursing theory and practice. Thus, nursing students may acquire a professional identity, consciously or unconsciously, during their professional education. Nursing educators are recommended to develop professional education programs and to promote the active participation of the students.

### Limitations

The limitations of this study include the non-generalizable nature of qualitative studies and the inclusion of participants from one nursing school only. Furthermore, the participants did not confirm the correctness of their information after transcription and thematisation and only two male nursing students joined this study as participants.
